# Crystal Structure of Outer Membrane Protein NMB0315 from *Neisseria meningitidis*

**DOI:** 10.1371/journal.pone.0026845

**Published:** 2011-10-26

**Authors:** Xiangyu Wang, Xue Yang, Chunting Yang, Zhenhua Wu, Honglin Xu, Yuequan Shen

**Affiliations:** 1 State Key Laboratory of Medicinal Chemical Biology, Nankai University, Tianjin, China; 2 College of Life Sciences, Nankai University, Tianjin, China; 3 Laboratory of Virology, National Vaccine and Serum Institute, Beijing, China; Institute of Molecular and Cell Biology, Singapore

## Abstract

NMB0315 is an outer membrane protein of *Neisseria meningitidis* serogroup B (NMB) and a potential candidate for a broad-spectrum vaccine against meningococcal disease. The crystal structure of NMB0315 was solved by single-wavelength anomalous dispersion (SAD) at a resolution of 2.4 Å and revealed to be a lysostaphin-type peptidase of the M23 metallopeptidase family. The overall structure consists of three well-separated domains and has no similarity to any previously published structure. However, only the topology of the carboxyl-terminal domain is highly conserved among members of this family, and this domain is a zinc-dependent catalytic unit. The amino-terminal domain of the structure blocks the substrate binding pocket in the carboxyl-terminal domain, indicating that the wild-type full-length protein is in an inactive conformational state. Our studies improve the understanding of the catalytic mechanism of M23 metallopeptidases.

## Introduction

*Neisseria meningitidis*, a commensal bacterium found in the human nasopharynx, has caused global health concerns owing to its potential to cause meningitis and sepsis. Pandemic outbreaks of meningococcal disease (MD), which have been reported worldwide, can be devastating, with a mortality rate of approximately 10% in many countries [1], [2]. Because of the rapid progress of the disease after its onset, early prevention seems to be indispensable in seeking a cure, leading to a global research effort to create a vaccine. The majority of MD is caused by 6 of the 13 serogroups of *Neisseria meningitidis*, as classified according to the polysaccharide capsule surrounding the bacterium [3], [4]. Despite a number of specific vaccines that have been developed against these pathogens, frequent antigenic variation hampers the generation of a broad-spectrum vaccine. The outer membrane proteins (OMPs) of *N. meningitidis*, which have been implicated in bacterial virulence, can effectively induce an immune response and thus are a good antigen candidate for vaccine design [5]. For such reasons, a number of OMPs have been identified and studied, including NMB0315, a putative metallopeptidase.

NMB0315 is an OMP identified in *Neisseria meningitidis* as belonging to serogroup B. The development of a universal vaccine against this group remains a challenge [6]. Although few studies have been dedicated to the uncharacterized protein NMB0315, sequence similarities suggest that it is a lysostaphin-type metallopeptidase [7], [8] belonging to the M23 peptidase family [9]. Proteins in this family are characterized by a conserved active site containing an HXH motif [8], [10], and all are zinc-dependent. They have been reported to have a hydrolytic specificity for a glycine-glycine peptide bond [11], [12], [13]. Over the past few decades, many members of the M23 metallopeptidase family have been identified, and fine structure analysis has been performed for some. Although the proteolytic mechanism of the conserved Zn^2+^-containing active site has been proposed and discussed, the diversity of the overall structures has resulted in confusion regarding their functional similarity in different bacteria [14], [15], [16]. Moreover, it has been reported that some of the Zn^2+^-dependent peptidases were synthesized as proenzymes and thus are inactive against the preferred substrates, such as polyglycine peptides [14], [17], [18].

In Gram-positive bacteria, such as *Bacillus subtilis*, lysostaphins are involved in the degradation of cell wall peptidoglycan during cell growth and separation [19], [20]. However, in Gram-negative bacteria, the peptidoglycan cell wall is directly crosslinked to each other, and thus, it lacks the glycine-rich linker targeted by these peptidases [21]. In *Pseudomonas aeruginosa*, LasA, which contributes to the virulence of *P. aeruginosa*, has been confirmed to be an M23 peptidase and implicated in elastin degradation [22]. Another Gram-negative bacterial M23 peptidase, the *Yersinia pestis* NlpD lipoprotein, has been shown not only to be required in cell division but also to serve as an essential virulence factor for bubonic and pneumonic plague development [23]. Moreover, mutant NlpD appeared to be a superior vaccine candidate that provided effective immunity, indicating the potential for members of the M23 peptidase family in Gram-negative bacteria to be used for disease prevention [23].

A BLAST sequence search showed that certain proteins with at least 98% sequence identity to NMB0315 were found not only in different serogroups of *N. meningitidis* but also in *Neisseria gonorrhoeae*, the other species of the genus *Neisseria* that is pathogenic in humans [24]. Although these two virulent species differ in niche preference and the nature of the induced disease, their use of the same survival or pathogenic mechanism based on NMB0315 is intriguing.

Here, we show the crystal structure of NMB0315 at 2.4 Å resolution. A metal-coordinating site in our structure resembles the previously reported structure of a Zn^2+^-containing metalloendopeptidase, indicating that NMB0315 is a member of the M23 peptidase family.

## Results

### Overall structure of NMB0315

The structure of NMB0315 was solved by single-wavelength anomalous dispersion at 2.4 Å resolution. Two molecules of NMB0315 were present in one asymmetric unit. In the final model, molecule A covers residues 57–95, 102–245, 250–262 and 266–420, whereas molecule B covers residues 58–245 and 251–420. The two molecules in the asymmetric unit have a root-mean-square deviation (RMSD) value of 0.7 Å for the 349 Cα atoms. We will refer only to molecule B in the following discussion.

**Figure 1:**
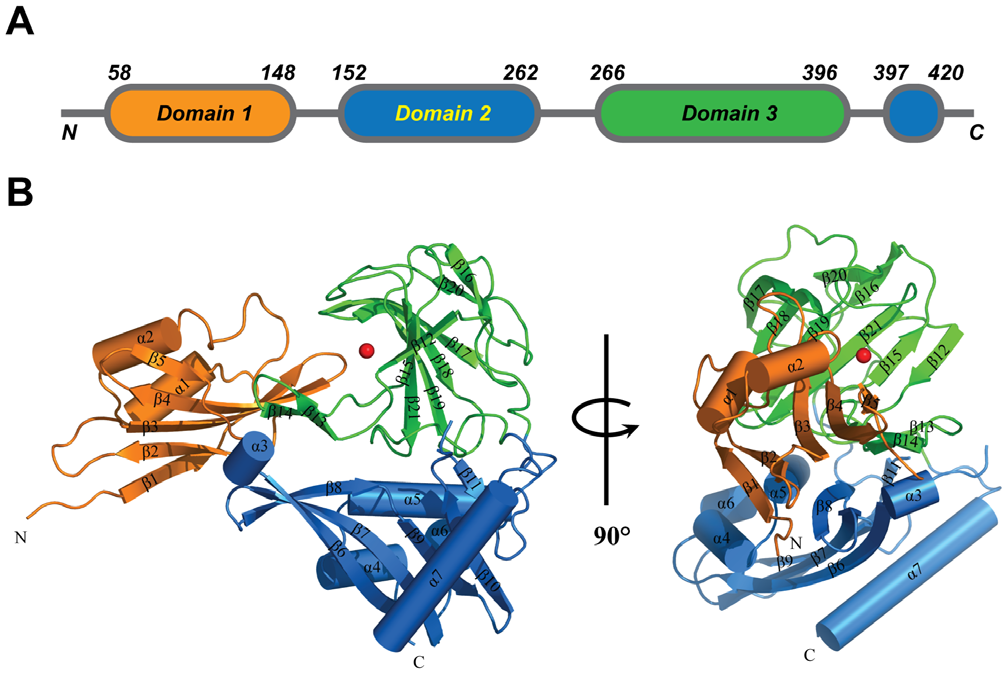
Crystal structure of NMB0315. (**A**) Overall topology of NMB0315. (**B**) Cartoon representation of NMB0315. The metal atom is shown as a red ball. Domains I, II and III are colored in orange, blue and green, respectively.

The overall structure consists of three spatially separated domains (Figure 1): Domain I (residues 58–151), Domain II (residues 152–265 and 397–420) and Domain III (residues 266–396). Domain I contains five anti-parallel β-strands (β1-β5) flanked by two α-helices (α1-α2). Domain II is made up of four α-helices (α3-α6), six β-strands (β6-β11) and one long C-terminal α-helix (α7). Domain III is solely formed by ten β-strands (β12-β21) (Figure 1). Two anti-parallel β-strands (β13 and β14) of Domain III extend out and interact with a β-strand of Domain I. The β3-β4 loop of Domain I points into the groove of the central β-strand region of Domain III.

### The active site

**Figure 2:**
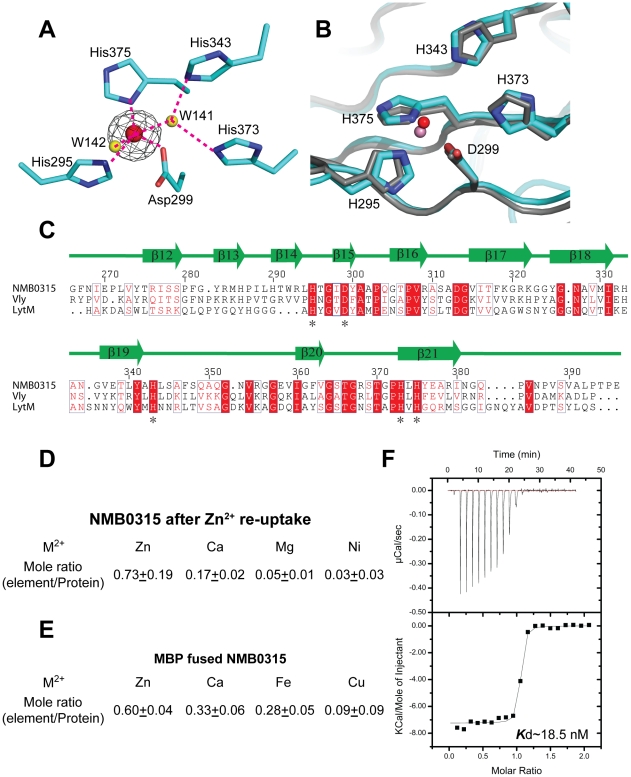
The catalytic pocket of NMB0315. (**A**) The metal ion binding site. Nitrogen, oxygen and carbon atoms are colored blue, red and cyan, respectively. The metal ion and water molecules are shown as red and yellow balls, respectively. The initial experimental electron density map calculated from the SAD phases is shown contoured at 14σ in black. Hydrogen bonds are shown as red dashed lines. (**B**) The superposition of the active site of NMB0315 onto LasA. The side chains of conserved residues are shown as sticks, and the main chain is shown as loops. NMB0315 is colored cyan, with the nitrogen and oxygen atoms of the side chains colored blue and red, respectively. LasA is colored gray. The metal ions of NMB0315 and LasA in the active sites are represented as red and light pink spheres, respectively. (**C**) Sequence alignment of NMB0315, Vly and LytM. Secondary structure elements of NMB0315 are shown as arrows (β-strands). The amino acids highlighted in red and denoted with asterisks are key residues in the active site that are conserved across the three proteins. (**D,E**) The atomic absorption spectrum for the different metal elements in the purified NMB0315 protein solution. (**F**) ITC measurement of the binding affinity between Zn^2+^ and NMB0315.

The 2***F***o-***F***c electron density map reveals a metal ion bound in domain III (Figure 2A). This metal ion is coordinated by three spatially adjacent residues (His295, Asp299, and His375) and two water molecules (W142 and W141). Two other histidines, His343 and His373, take part in interactions with the metal ion through the water molecule W141 (Figure 2A). This organization of the active site is highly conserved in M23 family members (Figure 2B). A sequence alignment also reveals that these five residues are highly conserved among M23 peptidases (Figure 2C). Because all peptidases of this family that have been found so far are zinc-dependent, we first searched for zinc in our structure. However, no signal was detected for a zinc atom in an X-ray fluorescence spectrum. One possibility is that the Zn^2+^ was replaced by another metal ion such as Ni^2+^ during the expression and purification of the protein.

To test this hypothesis, we treated the NMB0315 protein with EDTA to chelate bound metal ions and induce reuptake of Zn^2+^ into NMB0315 (for details, please see the methods section). The atomic absorption spectroscopy data clearly show that the mole ratio of Zn^2+^:protein is around 0.7, indicating that Zn^2+^ is included in the NMB0315 protein (Figure 2D). On the other hand, we purified Maltose binding protein (MBP) fused NMB0315 by MBP beads (GE healthcare) and Gel-filtration column. We found that the mole ratio of Zn^2+^:MBP-NMB0315 is around 0.6, confirming that Zn^2+^ can bind in the active site of NMB0315 (Figure 2E). To directly measure the binding affinity of Zn^2+^ with NMB0315, we titrated Zn^2+^ into NMB0315 protein pre-treated with EDTA using isothermal titration calorimetry (ITC). The calculated ***K***d value is ∼18.5 nM (Figure 2F).

### Structure comparison of NMB0315 and other M23 metallopeptidases

**Figure 3:**
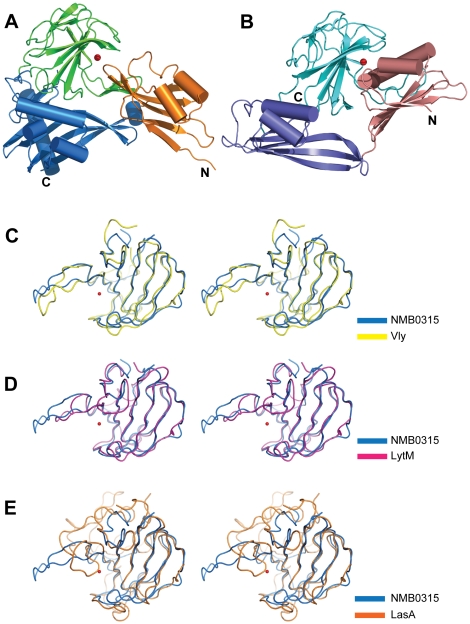
Structure comparison of NMB0315 with other M23 metallopeptidases. (**A**) Comparison of the overall structure of NMB0315 (left) and Vly (right). Each domain from one protein resembles the corresponding domain in the other; however, the spatial arrangements of the three domains between the two proteins are different. (**B**) Stereoview of the superposition of NMB0315 onto Vly (yellow), LytM (magenta) (**C**), or LasA (orange) (**D**).

A structural comparison based on entire structure of NMB0315 using the Dali Server [25] resulted in only one structure that is similar to NMB0315, a putative lysostaphin-type protein from *Vibrio cholerae* (referred to as Vly hereafter) (PDB ID: 2GU1). This three-domain containing protein belongs to the M23 peptidase superfamily and has been reported to be in an inhibited conformational state [16]. A side-by-side structural comparison of NMB0315 and Vly showed that the overall spatial arrangement of the three domains is distinct. However, the corresponding individual domains resemble one another (Figure 3A,B). This is especially true for Domain III, for which the comparison of these two domains is strikingly similar, with an RMSD value of 1.5Å for 112 Cα atoms (Figure 3C). Moreover, the three-dimensional structural features of Domain III are shared by many other proteins. More than 10 structural homologs of Domain III alone were found by the Dali Server, including the catalytic domain of LytM from *Staphylococcus aureus* (PDB ID: 2B13) (Figure 3D) and LasA from *Pseudomonas aeruginosa* (PDB ID: 3IT5) (Figure 3E). LytM is a zinc-dependent lysostaphin-type metallopeptidase involved in the autolysis mediated by *Staphylococcus aureus*, which is required for bacterial cell growth, separation and metabolism [26]. The structure of the active fragment of LytM has strong similarities to Domain III of NMB0315, but differences exist. A notable distinction between the two structures is the β12-β15 segment, which extends far from the β core in NMB0315 but is shorter in LytM (Figure 3D).

LasA is another M23 metallopeptidase known to be involved in entry to the host cell and other processes related to *Pseudomonas aeruginosa* virulence [22], [27], [28]. Like LytM, LasA shares a conserved β-strand region corresponding to the catalytic groove. However, it differs most in the C-terminus of NMB0315 Domain III, forming a three stranded β-sheet (Figure 3E) rather than an α-helix (Figure 3A).

### Auto-inhibition state of NMB0315

**Figure 4:**
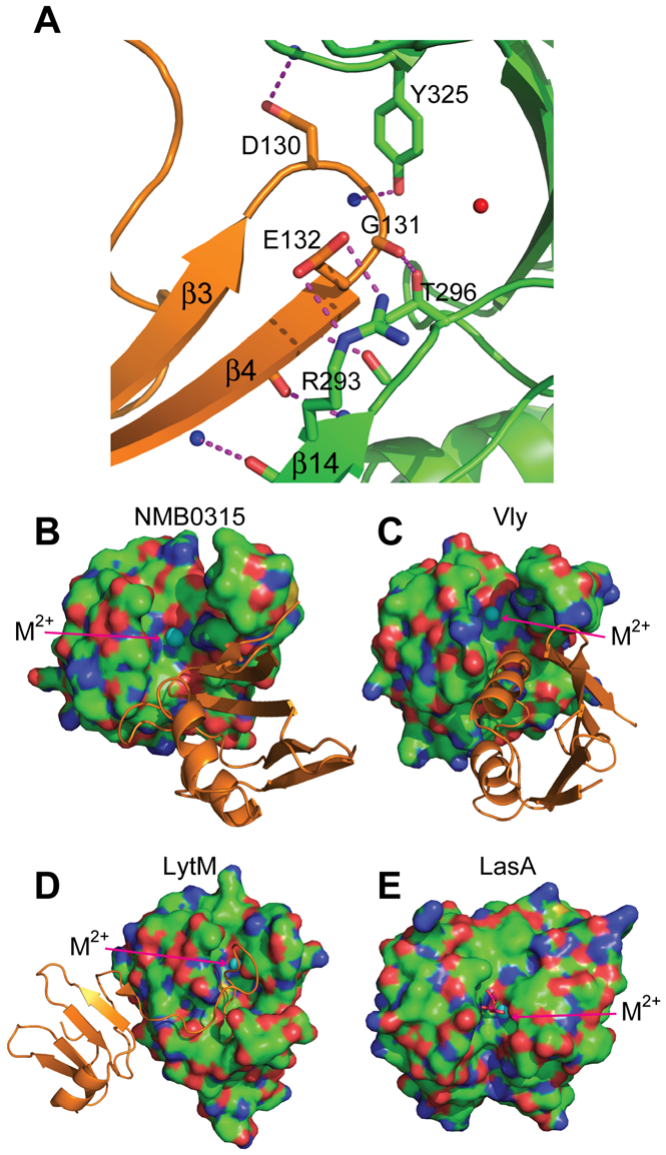
The auto-inhibition mechanism of NMB0315. (**A**) The inhibition loop of Domain I of NMB0315 is stabilized by numerous interactions with Domain III. The main chains of Domain I and Domain III are colored orange and green, respectively. The hydrogen bonds are shown as dashed magenta lines. The substrate binding grooves are blocked in NMB0315 (**B**), Vly (**C**), and LytM (**D**) but open in LasA (**E**). The catalytic domains of these four proteins are shown as surfaces in (**B**), (**C**), (**D**) and (**E**), and the inhibiting domains are shown as cartoons. The metal ions (M^2+^) in the active sites of these enzymes are represented as cyan spheres. A tartrate molecule is colored as magenta in the open catalytic site of LasA.

In our structure, the short β3-β4 loop of Domain I stretches into the catalytic center of Domain III and forms numerous interactions (Figure 4A). First, the β4 strand of Domain I forms an anti-parallel interaction with the β14 strand of Domain III, and they stabilize each other through several main-chain hydrogen bonds. Second, Glu132 of Domain I makes a salt bridge with Arg293 of Domain III. Third, the main-chain atoms of Gly131 of Domain I make two hydrogen bonds with two residues Tyr325 and Thr296 of Domain III. Fourth, Asp130 of Domain I forms a hydrogen bond with the carbonyl oxygen atom of Gly324 from Domain III. Based on these interactions, Domain I of NMB0315 is capable of tightly associating with Domain III, which blocks the active site and the substrate binding groove (Figure 4B), suggesting that full-length NMB0315 is in an auto-inhibited conformational state. This kind of inhibition seems to be quite different from other members of M23 metallopeptidase family, including Vly and LytM. The full-length Vly protein is also in a latent form, and the inhibition of its active site is achieved by an N-terminal helix from Domain I (Figure 4C). Wild-type LytM is also inhibited by its long N-terminal loop (Figure 4D) and strongly activated by the physiologically unrelated peptidase trypsin [29]. In contrast, the active site and substrate binding groove of the protein LasA are wide open and ready for substrate entry (Figure 4E).

## Discussion

Our results suggest that NMB0315 is a zinc-dependent peptidase and belongs to the M23 metallopeptidase family. A metal-containing active site was observed in our structure, in which one asparagine and two histidines are involved in the central metal ion coordination, with two additional histidines flanking one side. Given that this active site is highly conserved among members of this family, NMB0315 likely prefers glycine-containing peptides as its substrates. Remarkably, two water molecules, which were found to be involved in the coordination of the metal ion, bonded in the same way as that reported for the crystal structure of LasA [15]. A catalytic mechanism has been proposed based on the configuration of the active site of LasA. The incoming substrate peptide may take the position of one water molecule (corresponding to W142), with its peptide carbonyl bond polarized by the metal ion, and the other water molecule (corresponding to W141) likely functions as a proton donor, performing a nucleophilic attack on the carbonyl carbon and causing the hydrolysis of the peptide [15]. In the active site of our structure, both the residue arrangement and the two water binding sites resemble that of LasA, suggesting that a similar mechanism of catalysis may exist for NMB0315.

Interestingly, a short loop from Domain I of NMB0315 is positioned in the vicinity of the active site, apparently blocking peptide substrates. This intruding loop is further stabilized by interactions with several spatially adjacent residues (Figure 4A), which seemingly renders the peptidase constitutively inactive. This inhibition strategy differs from that of the other reported structures of M23 metallopeptidases, such as full-length LytM, which is self-inhibited by a loop from its N-terminus [14]. In the structure of Vly, an α-helix from the first domain, instead of a loop, prevents substrate access to the active site [16]. By contrast, despite the above differences, all three proteins share the conformational feature of using a segment of the N-terminus to block the active site. This evidence suggests that NMB0315, like other lysostaphins, is synthesized as a proenzyme and needs further processing to become active [29].

Based on the comparison of NMB0315 and other M23 metallopeptidases in their active form, we propose that the maturation of NMB0315 requires the amino-terminal domain to be removed. However, the truncated segment containing only domain III was very unstable and therefore had a low yield, which hindered any investigation into its catalytic activity. Further studies on NMB0315 and other peptidases from this family are expected to provide a more complete understanding of the inhibition, activation and hydrolytic mechanism of M23 metallopeptidases.

## Materials and Methods

### Protein expression and purification

The gene encoding residues 54–420 of NMB0315 protein were cloned into a modified pET-32a (Novagen) vector, in which the Trx tag was deleted by cleavage at two NdeI restriction sites and the thrombin site and the S tag were replaced by a prescission protease site (Leu-Glu-Val-Leu-Phe-Gln-Gly-Pro) and transformed into *E. coli* BL21 (DE3) cells. The resultant cells were then grown in LB medium at 25°C to A600 = 0.6, induced by isopropyl-1-thiogalactopyranoside (IPTG) at a final concentration of 0.2 mM and harvested after overnight incubation.

The cells were lysed in T_20_N_500_P_0.1_ buffer (20 mM Tris-HCl pH 7.0, 0.1 mM phenylmethylsulfonyl fluoride, 500 mM NaCl) by lysozyme (0.1 mg/ml) and sonication. After centrifugation at 20,000 g for 60 min, the supernatant was loaded onto a Ni-NTA column, washed with T_20_N_500_P_0.1_ buffer containing 20 mM imidazole and eluted with the same buffer to which 500 mM imidazole had been added. The eluted His_6_-tagged protein was digested by prescission protease at 4°C overnight and further purified by gel filtration on a Superdex G-200 column (GE Healthcare) in T_20_N_500_ buffer.

DNA fragment encoding residues 54–420 of NMB0315 was cloned into the pET-MBP vector (Novagen) and then transformed into BL21 codon plus *Escherichia coli* cells. The overexpressed protein lysate in T_20_N_400_ buffer (20 mM Tris-HCl, pH 7.0 and 400 mM NaCl) was loaded directly onto an MBPTrap HP column (GE Healthcare). After washing the column with 5 column volumes of T_20_N_400_ buffer, the MBP-tagged protein was eluted with T_20_N_400_ buffer containing 10 mM maltose. The fusion protein was loaded onto a HighLoad 26/60 Superdex-200 size-exclusion column (GE Healthcare) and eluted with T_20_N_400_ buffer at a flow rate of 2.0 ml/min. The protein peak was identified by SDS-PAGE gel and harvested and concentrated by Centricon (Millipore).

Seleno-methionine (Se-Met) labeled NMB0315 was expressed by *E. coli* B834 cells, which are methionine auxotrophs, in minimal medium under the conditions described above, and the derived protein was also purified following the protocol above.

### Crystallization and data collection

Crystals of either wild-type or Se-Met substituted NMB0315 were grown at 4°C at a protein concentration of 40 mg/ml using the sitting drop vapor diffusion method and equilibration against a reservoir solution of 7% PEG20,000, 0.1 M Tris-HCl, pH 8.5 and 2% 1,4-Dioxane. After growing for more than 5 days, the crystals were frozen in a cryoprotectant consisting of the reservoir solution supplemented with 25% glycerol.

Both the native dataset for the wild-type crystal and the single wavelength anomalous dispersion dataset for the Se-Met derivative were collected at the Shanghai Synchrotron Radiation Facility (SSRF). The latter was collected at the peak wavelength for Se atoms. The wild-type crystals of NMB0315 diffracted to 2.40 Å in the space group ***C***2 with unit cell dimensions of ***a*** = 195.68 Å, ***b*** = 75.50 Å, ***c*** = 81.64 Å and ***β*** = 94.66°. The Se-Met substituted crystals diffracted to 2.85 Å in the same space group and unit cell dimensions of ***a*** = 195.20 Å, ***b*** = 76.63 Å, ***c*** = 81.58 Å and ***β*** = 94.78°. Both datasets were processed and scaled with the HKL2000 software package [30].

### Structure determination and refinement

The program HKL2MAP [31] yielded seven of the eight theoretical Se sites in one asymmetric unit, and the initial SAD phases were calculated by PHENIX software [32]. The model was first built automatically by the PHENIX program package and then manually modeled using the COOT [33] program on the basis of 2***F***o–***F***c and ***F***o–***F***c difference Fourier maps. The structure model was refined using the CNS [34] program. The final structure had an ***R***_cryst_ value of 25.5% and an ***R***_free_ value of 26.4%. The Ramachandran plot calculated by PROCHECK [35] showed that 83.2% of the residues were in their most favored regions; 15.2% of the residues were in additionally allowed regions; 1.7% of the residues were in generously allowed regions; and no residues were in disallowed regions. Detailed data collection and refinement statistics are summarized in Table 1.

**Table 1:** Data collection and refinement statistics.

Crystal name	Wild-type	Se-Met-crystal
Space group	*C*2	*C*2
Unit cell (Å)	***a*** = 195.68, ***b*** = 71.50,***c*** = 81.64, ***β*** = 94.66°	***a*** = 195.20, ***b*** = 76.63,***c*** = 81.58, ***β*** = 94.78°
Wavelength (Å)	0.9796	0.9792
Resolution range (Å)	50–2.40(2.49–2.40)	50–2.85(2.96–2.85)
No. of unique reflections	38,289	25,693
Redundancy	6.0(4.4)^a^	3.7(2.8)^a^
***R***_sym_ (%)^b^	6.6(44.9)^a^	9.2(36.9)^a^
***I***/σ	24.6(2.4)^a^	17.0(1.9)^a^
Completeness (%)	99.5(50.5)^a^	99.2(46.1)^a^
**FOM**	---	0.685
Refinement		
Resolution range (Å)	37.75∼2.41	
***R***_crystal_ (%)^c^	25.5	
***R***_free_ (%)^d^	26.4	
RMSD_bond_ (Å)	0.007	
RMSD_angle_(°)	1.0	
Number of		
Protein atomsLigand atoms	5,3642	
Solvent atoms	0	
Residues in (%)		
most favored	494	
additional allowed	90	
Generously allowed	10	
disallowed	0	
Average B factor (Å^2^) ofProteinLigand atoms	84.9273.37	

a the highest resolution shell.

b
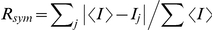

c ***R***_crystal_ = 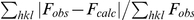

d ***R***_free_, calculated the same as ***R***_crystal_, but from a test set containing 5% of data excluded from the refinement calculation.

### Zn^2+^ reuptake and atomic absorption spectrum

To chelate metal ions in purified NMB0315, the protein (100 µM) was incubated with 10 mM EDTA at 4°C overnight. EDTA was then removed by loading the mixture onto a HiPrep 26/10 desalting column (GE Healthcare) and eluting the protein with T_20_N_500_ buffer. The Zn^2+^ reuptake was carried out by adding ZnSO_4_ manually to the protein sample to a final concentration of 5 mM and then incubating the mixture at 4°C overnight. The residual ZnSO_4_ was removed by passing the sample through a HiPrep 26/10 desalting column using T_20_N_500_ buffer.

To determine the atomic absorption spectrum, the 0.2 ml protein solution after Zn^2+^ reuptake was supplemented with 2 ml 65% nitric acid and incubated overnight. The sample was then diluted with double-distilled water and the concentrations of metal ions were determined by a polarized Zeeman Atomic Absorption spectrophotometer.

### Isothermal titration calorimetry

Measurements were carried out using an ITC-200 microcalorimeter (MicroCal) in T_20_N_400_ buffer (20 mM Tris-HCl pH 7.0, 400 mM NaCl) at room temperature. All samples were spun at 15,000 × *g* for degassing, and their concentrations were determined by both Bradford assay and spectrophotometry (280 nm wavelength). Sample solutions with a concentration of 0.05 mM were put into the sample cell, and the titration solution in the injection syringe was kept around 1 mM. To measure the binding constants, 20 consecutive injections of the titration into the calorimeter cell were collected at 120 intervals while being stirred at 1,000 rpm. The titration data were analyzed using MicroCal Origin software (MicroCal).

### Protein Data Bank and accession code

Coordinate and structure factor have been deposited in the Protein Data Bank with accession code 3SLU.

## References

[1] Hart CA, Rogers TR (1993). Meningococcal disease.. J Med Microbiol.

[2] Stephens DS, Greenwood B, Brandtzaeg P (2007). Epidemic meningitis, meningococcaemia, and Neisseria meningitidis.. Lancet.

[3] Frosch M, Maiden MC, Frosch M, Maiden MC (2006). The population biology of Neisseria meningitidis: implications for meningococcal disease, epidemiology and control.. Handbook of Meningococcal disease. Infection biology, vaccination, clinical management.

[4] Rosenstein NE, Perkins BA, Stephens DS, Popovic T, Hughes JM (2001). Meningococcal disease.. N Engl J Med.

[5] Pizza M, Scarlato V, Masignani V, Giuliani MM, Arico B (2000). Identification of vaccine candidates against serogroup B meningococcus by whole-genome sequencing.. Science.

[6] de Filippis I (2009). Quest for a broad-range vaccine against Neisseria meningitidis serogroup B: implications of genetic variations of the surface-exposed proteins.. J Med Microbiol.

[7] Schindler CA, Schuhardt VT (1964). Lysostaphin: A New Bacteriolytic Agent for the Staphylococcus.. Proc Natl Acad Sci U S A.

[8] Bochtler M, Odintsov SG, Marcyjaniak M, Sabala I (2004). Similar active sites in lysostaphins and D-Ala-D-Ala metallopeptidases.. Protein Sci.

[9] Rawlings ND, Morton FR, Barrett AJ (2006). MEROPS: the peptidase database.. Nucleic Acids Res.

[10] Hooper NM (1994). Families of zinc metalloproteases.. FEBS Lett.

[11] Ramadurai L, Lockwood KJ, Nadakavukaren MJ, Jayaswal RK (1999). Characterization of a chromosomally encoded glycylglycine endopeptidase of Staphylococcus aureus.. Microbiology.

[12] Sugai M, Fujiwara T, Akiyama T, Ohara M, Komatsuzawa H (1997). Purification and molecular characterization of glycylglycine endopeptidase produced by Staphylococcus capitis EPK1.. J Bacteriol.

[13] Barrett AJ, Rawlings ND, Woessner JF (1998). Handbook of proteolytic enzymes. San Diego: Academic Press..

[14] Odintsov SG, Sabala I, Marcyjaniak M, Bochtler M (2004). Latent LytM at 1.3A resolution.. J Mol Biol.

[15] Spencer J, Murphy LM, Conners R, Sessions RB, Gamblin SJ (2010). Crystal structure of the LasA virulence factor from Pseudomonas aeruginosa: substrate specificity and mechanism of M23 metallopeptidases.. J Mol Biol.

[16] Ragumani S, Kumaran D, Burley SK, Swaminathan S (2008). Crystal structure of a putative lysostaphin peptidase from Vibrio cholerae.. Proteins.

[17] Gustin JK, Kessler E, Ohman DE (1996). A substitution at His-120 in the LasA protease of Pseudomonas aeruginosa blocks enzymatic activity without affecting propeptide processing or extracellular secretion.. J Bacteriol.

[18] Kessler E, Safrin M, Gustin JK, Ohman DE (1998). Elastase and the LasA protease of Pseudomonas aeruginosa are secreted with their propeptides.. J Biol Chem.

[19] Blackman SA, Smith TJ, Foster SJ (1998). The role of autolysins during vegetative growth of Bacillus subtilis 168.. Microbiology.

[20] Smith TJ, Blackman SA, Foster SJ (2000). Autolysins of Bacillus subtilis: multiple enzymes with multiple functions.. Microbiology.

[21] Schleifer KH, Kandler O (1972). Peptidoglycan types of bacterial cell walls and their taxonomic implications.. Bacteriol Rev.

[22] Peters JE, Galloway DR (1990). Purification and characterization of an active fragment of the LasA protein from Pseudomonas aeruginosa: enhancement of elastase activity.. J Bacteriol.

[23] Tidhar A, Flashner Y, Cohen S, Levi Y, Zauberman A (2009). The NlpD lipoprotein is a novel Yersinia pestis virulence factor essential for the development of plague.. PLoS One.

[24] Schielke S, Frosch M, Kurzai O (2010). Virulence determinants involved in differential host niche adaptation of Neisseria meningitidis and Neisseria gonorrhoeae.. Med Microbiol Immunol.

[25] Holm L, Sander C (1995). Dali: a network tool for protein structure comparison.. Trends Biochem Sci.

[26] Ramadurai L, Jayaswal RK (1997). Molecular cloning, sequencing, and expression of lytM, a unique autolytic gene of Staphylococcus aureus.. J Bacteriol.

[27] Park PW, Pier GB, Preston MJ, Goldberger O, Fitzgerald ML (2000). Syndecan-1 shedding is enhanced by LasA, a secreted virulence factor of Pseudomonas aeruginosa.. J Biol Chem.

[28] Park PW, Pier GB, Hinkes MT, Bernfield M (2001). Exploitation of syndecan-1 shedding by Pseudomonas aeruginosa enhances virulence.. Nature.

[29] Firczuk M, Mucha A, Bochtler M (2005). Crystal structures of active LytM.. J Mol Biol.

[30] Otwinowski Z, Minor W (1997). Processing of X-ray diffraction data collected in oscillation mode.. Methods Enzymol.

[31] Pape T, Schneider TR (2004). HKL2MAP: a graphical user interface for macromolecular phasing with SHELX programs.. J Appl Crystallogr.

[32] Zwart PH, Afonine PV, Grosse-Kunstleve RW, Hung LW, Ioerger TR (2008). Automated structure solution with the PHENIX suite.. Methods Mol Biol.

[33] Emsley P, Cowtan K (2004). Coot: model-building tools for molecular graphics.. Acta Crystallogr D Biol Crystallogr.

[34] Brunger AT, Adams PD, Clore GM, DeLano WL, Gros P (1998). Crystallography & NMR system: A new software suite for macromolecular structure determination.. Acta Crystallogr D Biol Crystallogr.

[35] Laskowski RA, MacArthur MW, Moss DS, Thornton JM (1993). PROCHECK: a program to check the stereochemical quality of protein structures.. J Appl Crystallogr.

